# Geographic variation in selected hospital procedures and services in the Israeli health care system

**DOI:** 10.1186/s13584-016-0127-y

**Published:** 2017-01-16

**Authors:** Joseph Mendlovic, Ethel-Sherry Gordon, Ziona Haklai, Jill Meron, Arnon Afek

**Affiliations:** 1 Ministry of Health, Israel, Jerusalem, 93722 Israel; 2 Shaare Zedek Medical Center, Jerusalem, Israel; 3 Sackler school of medicine, Tel-Aviv University, Tel-Aviv, Israel

**Keywords:** Geographic variations, PTCA, Periphery, Ministry of Health

## Abstract

**Background:**

Medical practice variation refers to differences in health service utilization among regions in the same country. It is used as a tool for studying health inequities.

In 2011, the OECD launched a Medical Practice Variation Project which examines regional differences within countries and explores the sources of the inter-regional differences. The aim of this study is to examine the patterns and trends in geographic variation for selected health services in Israel.

**Methods:**

The analysis is based on data from the National Hospital Discharges Database (NHDD) of the Israeli Ministry of Health. The eight procedures and services studied were: medical admissions (i.e. admissions without surgical procedures); hip fractures; caesarian sections; diagnostic cardiac catheterization; cardiac angioplasty (PTCA); cardiac bypass surgery (CABG); hysterectomy; and knee replacement surgery. The data are presented for the 7 districts in Israel, determined by address of residence.

**Results:**

The procedures and services with the lowest variation across the seven districts were medical admissions (RR between regions-maximum/minimum 1.3) and hip fractures (RR 1.44), while the one with the highest variation was CABG (RR 1.98). The Israeli periphery, and the northern district in particular, had higher rates of medical admissions, knee replacement and cardiac procedures. When studying the trend over time, we found a decrease in use rates for most procedures, such as coronary bypass (R. 04) and CABG (R 0.8). Medical admissions decreased by 8%, with the highest decline (16%) observed in the central districts.

**Conclusions:**

This study provides Israeli policy makers with information which is vital for the strategic planning of service development, such as strengthening preventive medical services in the community, reducing cardiovascular risk factors in the periphery and expanding the national publication of clinical quality scores.

**Electronic supplementary material:**

The online version of this article (doi:10.1186/s13584-016-0127-y) contains supplementary material, which is available to authorized users.

## Background

In recent years, much effort has been expended by the Israeli government to reduce the gaps between the country’s periphery and center in the health sector. Internationally, one of the first and most prominent tools in this area that compares variations in health services between different areas is called Medical Practice Variations. The first research group in this field published its work in what later developed to be known as “medical practice variation studies” or “small area analysis”^1^ and it was the first to demonstrate differences in the supply of health services between geographic regions in the U.S. The conclusions of this team were first published in 1973 and showed great differences in the consumption and supply of medical services between neighboring regions within the state of Vermont [1]. Since the 1970’s, the “Medical Practice Variations” tool has developed consistently and it is used as one of the main measures for evaluating gaps in the suplley of healthcare services. The OECD used this tool in 2011 for the “Medical Practice Variations” project which aims to examine regional differences within countries [2].

Typically, in countries with low inter-regional variation, services are considered to be optimized. However, achieving low medical variation doesn’t represent always good medical services (e.g. were low variation of effective care represent uniformly and nationally poor care). It is easier to achieve low medical variation in medical conditions with a simple diagnosis or treatment, such as treatments of fractures, as opposed to conditions requiring awareness and/or targeted testing, such as the treatment of colorectal cancer [3–5].

Large variations are often characterized by differences in socioeconomically status, and differences in the health condition of the population. In these cases there is a direct correlation between the use of health services and several influential factors such as resource allocation, medical staff employment, expertise, training and more [6–8].

In September 2014 the OECD published a report that examined the extent of inter-regional variation within 13 countries)Australia, Belgium, Canada, the Czech Republic, Finland, France, Germany, Italy, Portugal, Spain, Switzerland, England and Israel), for a variety medical service [9]. This article presents data and the conclusions of the research carried out by the Israel Ministry of Health, which were analyzed as part of the OECD medical variation project.

### Aim of the study

The aim of this study is to examine trends over the last decade in the use of selected hospital services by the population residing in Israel and to compare usage rates and trends across regions.

## Methods

The data presented in this study are based on the National Hospital Discharges Database (NHDD) maintained by the Health Information Division in Israel’s Ministry of Health. The database is continually updated, with hospitals providing information electronically on a quarterly basis. The database includes all acute care hospitals as well as some of the psychiatric and long-term care facilities. Only acute care hospitals were included in this study. The database contains records of each individual admission. Patients’ identity numbers are encrypted to allow follow-up studies, while preventing identification of individuals to protect patient privacy. The database includes demographic and hospitalization data. The demographic data include age, gender and residence (village/town/city code), as well as the patient’s health fund provider. The hospitalization data include general information such as admission type (planned or via the emergency room), discharge type (home, transfer to other facility or died), detailed information at the departmental level such as date of admission and discharge from each department, diagnoses listed and procedures performed in each admission, which are coded according to the ICD-9-CM classification.

Eight categories of hospital care were examined, after being selected by an OECD expert: medical admissions (i.e. admissions without surgical procedures^2^), hospitalization for hip fracture, caesarean sections, diagnostic cardiac catheterization, cardiac angioplasty therapy (PTCA), cardiac bypass surgery (CABG), hysterectomy and knee joint replacement surgery. The OECD expert group selected these care activities and procedures, based mainly on the criteria of high-cost and high-volume, policy relevance and data availability. The set of care activities and procedures included a general measure of hospital medical admissions, and specific diagnostic and surgical procedures.

The data are presented according to the seven districts in Israel, as defined by the Ministry of Interior. Smaller regions than the seven administrative districts are not feasible in Israel. Using sub-districts or cities would lead to too small volumes in certain districts for some procedures. The location was determined by the patients’ residence, and not the providing hospital location; the two differ because services are often provided in regions other than the region of residence. Tourists and other non-residents were excluded from the analysis.

Discharges by type of service are shown for the years 2002–2012. The identification of procedures is based on OECD guidelines (Additional file 1).

Age/gender-standardized rates were calculated by type of service and region and were generally presented as rates per 100,000 persons. The exception is the Caesarean rates, which were calculated per 1,000 live births and validated against the National Perinatal Database.

## Results

We studied the extent of regional variation in 2012, as well as national and regional trends over the period 2002–2012.

### Variations in procedure incidence between districts

**Fig. 1:**
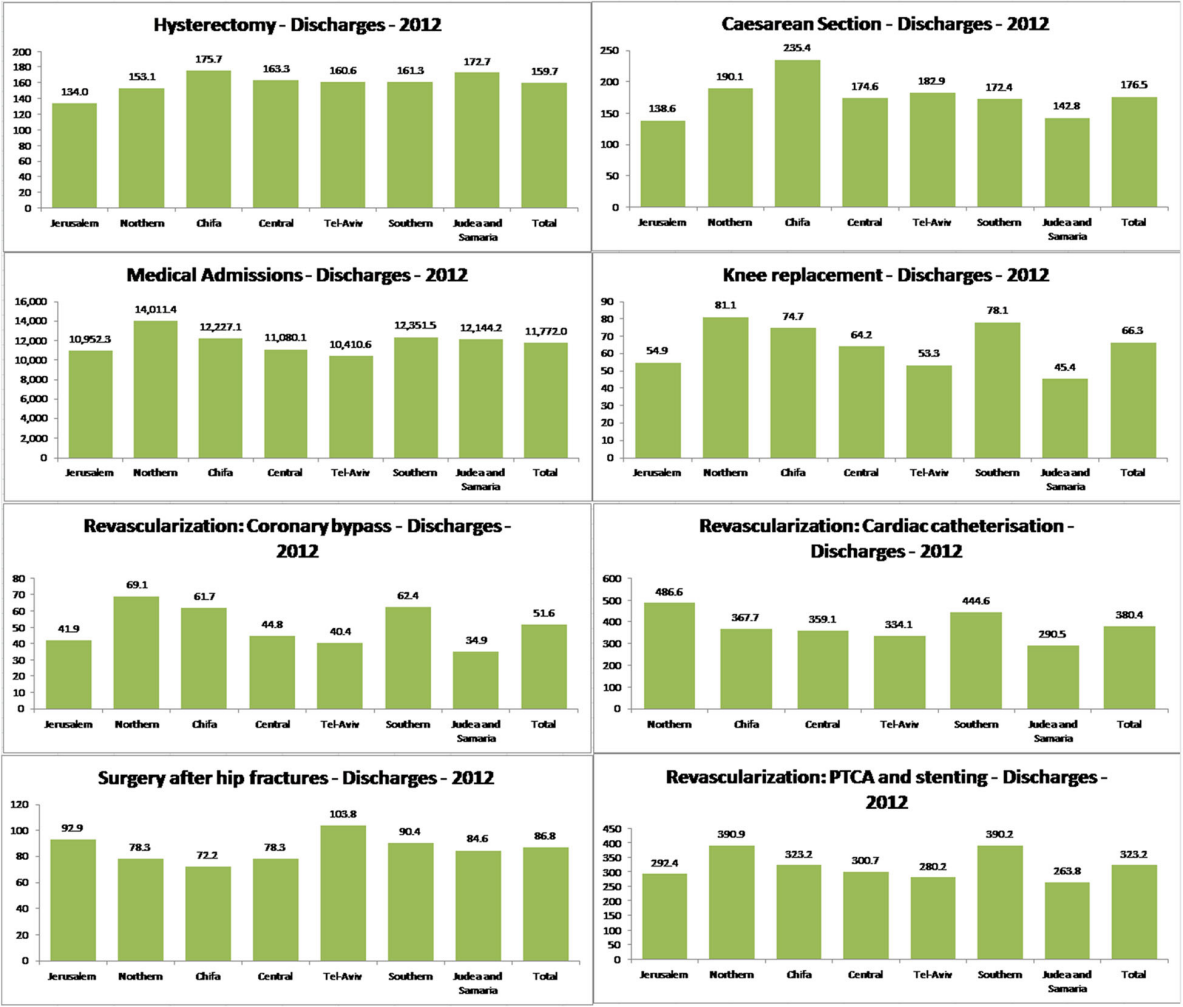
Medical services rates by district, 2012. (Age and gender adjusted rates per 100,000 persons)

Table 1 displays the adjusted rate, the coefficient of variation and the rate ratio (RR) between the highest and lowest regional rates. The data indicate differences between regions for all eight health care activities and procedures tested, with the relative rates (RR) of 1.3 or higher for all of them. Variation was highest for coronary bypass, with an RR of 2.0 between the highest and lowest regional rates, and knee joint replacement surgery with a RR of 1.8. In both these cases, the highest rates were in the Northern district. Figure 1 displays the medical services rates by district during 2012, adjusted for age and gender per 100,000 persons.

**Table 1:** Summary of the gaps for various hospital medical services in Israel by district, 2012

	Hysterectomy	Caesarian section^b﻿^	Knee Replacement	Surgery after hip fracture	Cardiac Catheterization	PTCA^c^	CABG	Hospital Medical Admissions
Standardized rate to −100,000 population^a^	159.7	176.5	66.3	86.8	380.4	323.2	51.6	11,772.0
Coefficient of variation between regions^d^	0.09	0.18	0.21	0.13	0.19	0.16	0.26	0.10
Ratio between regions (maximum /minimum) - RR	1.31	1.70	1.78	1.44	1.68	1.48	1.98	1.35

^a^Rates are standardized for age and gender to 100,000

^b^Cesarean rate is per 1,000 live births

^c^Percutaneous transluminal coronary angioplasty

^d^The coefficient of variation is the ratio of the standard deviation to the mean and is a systematic component of variation

### Trends in procedures over time

Examining trends in procedures and hospitalizations over the last decade indicates that the most significant increase was observed in knee joint replacement surgery, with an increase of more than half, from 42 per 100,000 persons in 2002 to 66 in 2012. The Southern district, Haifa district and the Northern district, recorded the highest growth in 2002–2012, resulting in the highest rate in 2012 (Table 2).

**Table 2:** Rates of 8 procedures between the years 2002 and 2012

	Adjusted rates a year (100,000)		
Activity	2002	2012	Ratio
Caesarean Section	175.6	176.5	1.0
Hysterectomy	187.9	159.7	0.8
Knee replacement	41.8	66.3	1.6
Medical Admissions	12,859.7	11,772.0	0.9
Revascularization: Cardiac catheterisation	489.5	380.4	0.8
Revascularization: Coronary bypass	122.1	51.6	0.4
Revascularization: PTCA and stenting	411.7	323.2	0.8
Surgery after hip fractures	97.5	86.8	0.9

Over the last decade, the hysterectomy rate decreased by 15%, from 188 per 100,000 females in 2002 to 160 in 2012.

The Caesarean section rate increased in 2002–2012 mainly in the Haifa district, and had almost 30% increment between these years.

**Fig. 2:**
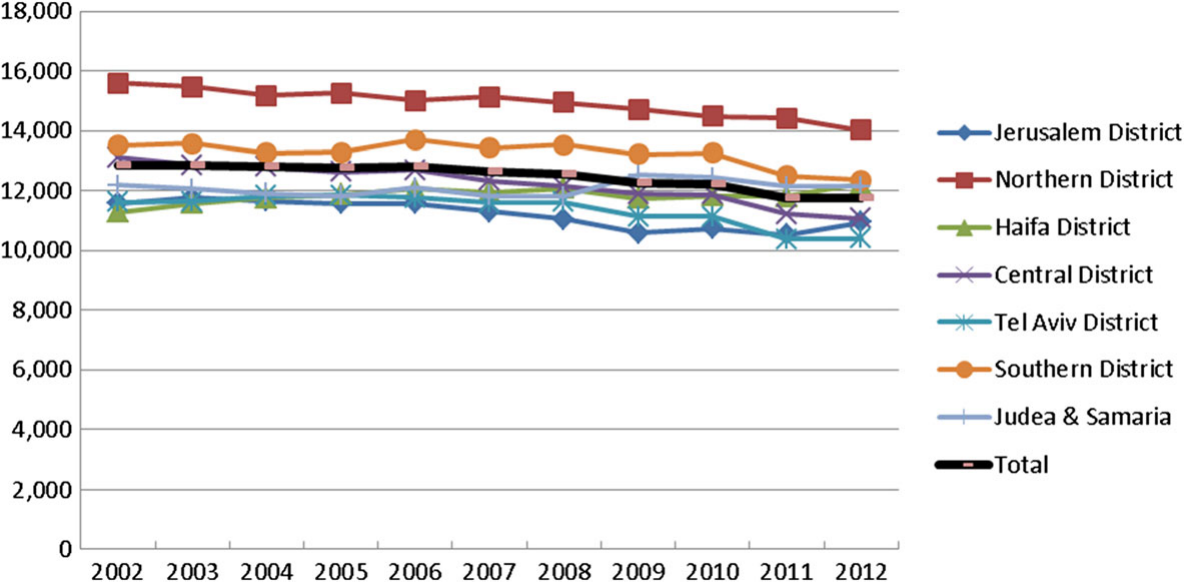
Medical admissions rate by districts, 2000–2012, adjusted for age and gender per 100,000 persons

The rate of medical admissions (Fig. 2) declined in all districts, with an 8% decline at the national level, and with the highest percentage decrease (16%) recorded in the Central district. Over the past decade, there was a much higher rate in the Northern and Southern districts compared to other regions.

Hospitalization rates for hip fracture repair decreased by 11% over the last decade, from 97 per 100,000 inhabitants in 2002 to 87 in 2012. In 2012, the highest rate was recorded in the Tel Aviv district with 104 per 100,000 inhabitants, 1.2 fold higher than the national average and 1.4 times higher than the Haifa district with the lowest rate of 72 per 100,000 inhabitants.

The rate of bypass surgery (CABG) plummeted by more than half, from 122 per 100,000 in 2002 to 52 in 2012. In most districts the rate decreased by two-thirds, while in the Northern and Haifa districts it decreased by 45%. In 2012, the highest rates of CABG were recorded in the Northern, Southern and Haifa districts.

The rate of cardiac catheterization (diagnostic) has decreased over the past decade. The national rate fell by 23% in 2002–2012, from 489 per 100,000 inhabitants to 380. The downward trend was recorded in most regions, with the largest decline (33%) in the Tel Aviv district, while in the Southern district the rate inclined in about 8% stable in the last decade

**Fig. 3:**
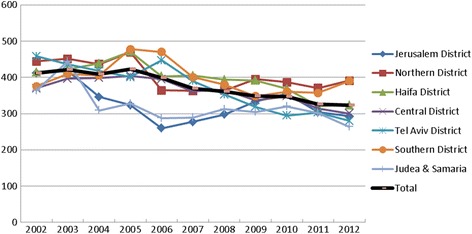
Percentage of therapeutic catheterizations (PTCA) rates by districts, 2002–2012, adjusted for age and gender per 100,000 persons. Diabetes and diabetes, and after first coronary event

The rate of therapeutic catheterization (PTCA) (Fig. 3) has decreased over the past decade. The national rate fell by 22% in 2002–2012, from 412 per 100,000 inhabitants to 323. The downward trend was recorded in most regions, with the largest decline (39%) in the Tel Aviv district, while in the Southern district the rate inclined in about 33% in the last decade. In the field of cardiac catheterization, the results reveals interesting phenomenon, both regarding the national negative trend, but especially when this trend is being studied in the light of the different regions rates. In 2012, the PTCA rate was highest in the Northern and Southern districts, 1.2 fold higher than the national average, while in other districts the rate was low compared with the national average. The highest rate for PTCA was 391 per 100,000 persons in the Northern district and 390 in the Southern district, fifty percent higher than the rate recorded in the Judea and Samaria which had a rate of 264 per 100,000 persons, and 1.4 times higher than the Tel Aviv district rate of 280, and 1.3 times higher than Jerusalem district rate of 292.

### International comparisons

For most of the health care services and procedures studied within the Geographic Variations in Health Care OECD report, the general trends in health care activities and procedure rates at the national level in Israel, over the past ten years, have been similar to those observed in many other OECD countries [ 9 ]. However, both trends and rates of PTCA in Israel are unique. Unlike trajectories in most other countries, which increased their rates, Israel has decreased its rates. However, even after this decrement, Israel still has one of the highest PTCA rates (along with Germany), while Portugal and Spain had the lowest rates (below 140 per 100 000).

## Discussion

This study analyzed both 2012 rates and 2002–2012 trends, for Israel as a whole and for its seven districts. For all of the services examined in this study, the 2012 rates were significantly different between the districts. The smallest differences between districts in 2012 were recorded for hysterectomies, with a rate ratio of 1.3, and the highest rate ratio was for CABG, with a rate ratio of 2.0.

Geographic variation in medical practice styles reflect not only differences in patient needs but might be due variations in practice of medicine, in resource allocation and in equal access to services. These unwarranted variations need to be addressed by countries who wish to improve healthcare performances. As the Darmoth project stats unwarranted variation in the practice of medicine and the use of medical resources are the inappropriate use of medical resources are the basis of ineffective care and must be addressed.^1^ This paper focuses on geographic variations in Israel so to learn and improve policy making. Usually, these unwarranted variations in the practice of medicine and the use of medical resources are being analyzed through several main general causes, among them: disease patterns among districts, degree of agreement between providers and supply-side factors, underuse of effective care, misuse of preference-sensitive care and overuse of supply-sensitive care. In order to provide a systemic approach for the policy makers in Israel, this paper will analyze the results via some of these methods.

With regard to the trends over time, at the national level, the study found a significant increase in knee replacement surgery, and significant declines in hip fracture repair surgery and hysterectomy. A decline in medical admissions was observed as well among all the seven regional trends. However, despite this decline there is still a consistent and clear gap between central and peripheral regions, reflecting the highest medical admission rate in the periphery and the lowest in the center of country.

In this section, we first consider possible explanations for the 2012 inter-regional differences and then consider possible explanations for the difference in trends across regions and services. We also discuss the extent to which the Israeli patterns and trends are similar to those found in other countries.

### Possible explanations for the inter-regional differences

Some of the inter-regional differences in 2012 rates may be related to uneven access to care as well as differences in socioeconomic and health status between the regions. Although these differences had never been published through geographical areas, they were studied and published by the Ministry Of Health report on 2013 through socioeconomic perspectives.^3^ Apparently, this Ministry of health 2013 report, demonstrated a significant difference both in obesity, smoking and in ischemic heart disease and had shown that low socioeconomic status have a direct linkage to these three risk factors and diseases. The extent to which rates, for particular procedures, vary across regions may also be related to the extent to which the relevant medical conditions can be prevented or controlled non-surgically. However, there isn’t any established explanation to the scale of all the variations in the different procedures. In the following paragraphs, we shall discuss the 5 procedures with the highest and lowest variations, by examining them systematically from three perspectives:

A) The disease patterns among districts B) The degree of agreement between providers C) The supply-side factors.

In addition, we shall discuss, when it is relevant, the results from additional perspectives: failure to provide effective care, failure to provide preference sensitive care, and overuse of supply sensitive care.

PTCA: The procedure which holds the highest variation with rate ratios of 1.98, in 2012, between the highest and lowest regional rate, is highest in the periphery (the northern and southern districts). Regarding the disease patterns, there is a direct correlation between ischemic heart diseases and the cardiovascular risk factors that are the highest in low socioeconomic status as mentioned earlier. Therefore, these differences are expected and will not change dramatically until there will be a reduction in smoking and obesity rates in the periphery. However, the high rates of CABG in the periphery could not be explained through the arguments of “degree of agreement” and “supply-side factors. This assumption derives from the fact that, the northern district, doesn”t hold a cardiac surgery unit, and the southern district holds only one.

This major variation correlates also to the underuse of effective care, as preventive medicine and glycemic control and also correlates to the Misuse of preference-sensitive care, in this case, cardiac catheterization (although the last one had increased dramatically in the last years).

Knee replacements, a procedure which holds the second highest variation with rate ratios of 1.78, in 2012, between the highest and lowest regional rate, is highest in the periphery. Regarding the disease patterns, this can be attributed to both the high rate of obesity and the lack of proper prevention in the periphery, which can be reduced by good obesity control and osteoarthritis treatment. Regarding the argument of “degree of agreement” there isn’t a clear indication for knee replacement, which can imply for an over use to this surgery in the periphery. Regarding “supply-side factors”, it is unreasonable that this is one of the reasons since most of the hospitals in the center of Israel hold several advances units, which specialized in knee replacement, where these units in the periphery are less developed.

This variation correlates also to the misuse of preference-sensitive care, in this case, conservative therapy such as physical therapy and weight loss.

The third procedure with the highest variation is cesarean section with rate ratios of 1.68, in 2012, between the highest (The city of Haifa) and lowest regional rate (The city of Jerusalem). This low rate in Jerusalem can be attributed to demographical and cultural reasons: In Jerusalem, which holds the lowest rates, the population is religious and therefore avoid Cesarean Section in order to reach high number of labors. In Chifa, which holds the highest rates, the population is secular with low rates of labors per capita.

This variation correlates also to the misuse of preference-sensitive care, in this case, a regular labor, and also overuse of supply sensitive care, since cesarean section is considered to be a convenient solution in difficult labors.

Hysterectomy, this procedure holds the lowest variation with rate ratios of 1.31, in 2012, between the highest and lowest regional rate.

Regarding the disease patterns, there are not preventable risk factors for the clinical statues that cause the need for this procedure. This fact minimizes the gaps between the periphery and the center of Israel in the usage of this procedure and implies on the importance of gaining equality, not only in treatment, but also in preventive medicine. Regarding the arguments of “degree of agreement”, there is not always clear indication for this procedure but there are still several common well established indications for the procedure. As for “supply-side factors”, there isn’t any specific uniqueness regarding the tariff or pricing method of this procedure. Therefore, the fact that the risk factors are uniform nationally could be a proper explanation to the low variation.

The second procedure with a relatively low variation is surgery after hip fracture, with rate ratios of 1.44, in 2012, between the highest (The city of Tel-Aviv) and lowest regional rate (The city of Chifa). This fracture is common especially in old people and females. Regarding the disease patterns, there are clear preventable risk factors for a hip fracture, among them preventing and treating osteoporosis. However, after stratifying the population to age and gender, there is no clear correlation in this aspect, since the highest rates are not in the periphery. Regarding the argument of “degree of agreement” there is a clear indication for surgery after hip fracture5, which can well explain the low rate of variation. As for “supply-side factors”, this relatively low variation could be explained by close control of the ministry of health on hospital. This control aims to measure the delivery of this surgery in 48 h for patients who were admitted to hospital with a hip fracture. The outcomes of this control are reflected both on a national publication of the results and also in a differential Tariff for hospitals which operating this patients in less than 48 h after admission. It is suggested that this policy, had led all hospitals to acquire similar guide lines and protocols for treating patients with this diagnosis.

Following these results and analysis, it is clear that the appropriate community medicine, before reaching hospital, is crucial for reducing variations between different regions, especially between the periphery and the center. Hence, it is not surprising that a 2010 study had revealed significant gaps in the availability of secondary community-based medical services, with relative shortages in the periphery for internal medicine, surgery and various sub-specialties [10]. This is consistent also with another finding of our research, which demonstrates higher rates of medical admissions in the periphery (with rate ratios of 1.34, in 2012, between the highest and lowest regional rate). The problematic availability of secondary community medical services in the periphery may have contributed to the transfer of activities from the community to hospitals, increasing the rate of hospital admissions. Interestingly, a related study found that gaps between the periphery and the center were also found in death rates [11].

### Possible explanations for the trends over time through local and international perspective

Generally speaking, the trends in health care activities and procedure rates at the national level in Israel over the past ten years have been similar to those observed in many other OECD countries.

Comparing trends in procedures and hospitalizations over the years 2001–2012 reveals that Israel has in most cases the same trends as the other countries that were measured in this study [9]. There has been a general reduction in hospital medical admissions, in surgery after hip fracture and in hysterectomy rates. There has been a substantial increase in knee replacement and caesarean section rates. These findings are mostly relevant to the procedures with clear positive or negative trends and could be explained by the same reasons: The most significant increase was observed in knee joint replacement surgery, with an increase of more than half. This extremely positive trend could be easily explained by the fact that widespread usage of this technology is quite new. The second most positive trend is for caesarean sections, which could be attributed to the expanding demand from patients for this procedure, and the expanding phenomenon of defensive medicine. The decline in hysterectomy could be attributed to the relatively clear guidelines and indications for this procedure. The decline in medical admission rates could be mostly attributed to the improvement of medical services in the community, an improved prevention and more accurate reporting policy.

The only procedure for which Israel (along with Italy) doesn’t converge to the general international trend is PTCA [11]. This phenomenon may be related to the fact that, in the early 2000’s, Israel used to have a very high rates relatively (Maximum of 401/100,000 population in 2003), which at that time was one of the highest rates in the OECD countries. Subsequently, this rate gradually converged to the prevailing international levels (293/100,000 population in 2011). As for an explanation to this relative high rates, especially in the early 2000’s but also later, one option could be attributed to the fact the tariff of PTCA was relatively very high In Israel, and therefore could have led to a high availability and performance via the mechanism Of supply induced demand. Only lately, (July 2013) the tariff had declined, but it is still relatively high, such as the rates of this procedure in Israel.

The trends regarding cardiac care procedures are mixed and difficult to interpret. On the one hand, as in many other OECD countries, there has been a sharp decline in CABG rates in Israel, as a higher share of people with ischemic heart disease were treated with less invasive procedures. These interventions where based on cardiac catheterization and aggressive preventive treatments which included both secondary prevention and primary, both in hyperlipidemia, pre-diabetes and diabetes, and after first coronary event.

On the other hand, the national standardized rate of coronary angioplasty (PTCA) also decreased between 2000 and 2011, which is not consistent with the trend observed in most other OECD countries.

### Israel’s inter-regional variation in international perspective

As national trends, the Israeli patterns of cross-regional variation are similar to those found in other countries. Knee replacement rates display high levels of variations, which can vary by more than fourfold across countries. Cardiac procedures rates show the highest level of geographic variations; they vary by more than three-fold across countries and also have the highest level of within-country variation for more than half of the countries.

However, analyzing Israel’s PTCA trends, which are not congruent with international trends of PTCA [11], raises the need to analyze this phenomenon via analyzing trends in CABG performance trends and regional trends as well.

On the one hand, as in most of the OECD countries, a sharp drop in the performance of coronary artery bypass surgery was seen in recent years. This decline is attributed to the diversion of some surgical activities into catheterization procedures. On the other hand, and unlike other OECD countries, a decline in the performance of invasive cardiac catheterization (PTCA) was observed. However, this downward trend was not uniform to all of the districts. While in the peripheral areas (the Northern and Southern districts), there was a sharp rise in the performance since 2002 to 2005 and from then some moderate changes, in other districts a decline was observed throughout the decade. These opposite trends, which can be seen in Fig. 3, have inversed the PTCA regional procedures rates from 2002 to 2012 and should be investigated separately, as they could be attributed to a the implications of “periphery reinforcement” policies. (see Additional file 2).

### Recent government activities to address the inter-regional differences

A thorough report published by the Ministry of Health in 2012 set a quantitative target of narrowing by 15% the disparities in mortality from cardiovascular conditions before age 75 between low-income persons and the population-wide average [12]. Given the fact that time and distance considerations are critically important in the case of a heart attack requiring urgent catheterization [13 14 ] the improvement in accessibility in this field has crucial importance in reducing gaps. It should be noted that the speed of performing cardiac catheterization in acute myocardial infarction is one of the quality measures that are examined by the Israeli Ministry of Health.^4^

The Ministry of Health, as part of a broader governmental policy, is continuing to strengthen the periphery by adding hospital beds and qualified personnel (residents and experts that since 2011 got a special bonus when choosing to work in the periphery), improving infrastructure and by adding quality measurements.

In the years ahead, the Ministry of Health will further investigate these trends in geographic variations in the use of hospital services across Israel and try to determine the factors that may explain these differences.

### Policy implications

The main policy implications of the findings of this study are as follow:

1. Steps should be taken to preserve the trend of reduced hospitalization rate by strengthening both community and preventive medicine services. These steps should be taken especially in the periphery, were the admission rates are still relatively high.
2. As the trend in PCI rates differs between the central of Israel and its periphery, and this is at variance with the situation in other countries, the Ministry of Health should carry out research which will examine these differences and their practical and clinical motives and implications.
3. As for the relatively high rates of the PCI procedure in Israel, the Ministry of Health should examine its high tariff.
4. Following the negative trend and low variation in hysterectomy rates, that are being attributed to the usage of clinical guide lines and to the fact that socioeconomic status doesn’t affect the risk factors that are leading to this procedure, the Ministry of Health should focus on reducing risk factors which derives from inequality in health and should consider expending the usage of clinical guidelines to other clinical fields [15].
5. Following the low variation in surgery after hip fracture, that are being attributed partially both to the national publication of the results and to the allocation of a differential tariff for hospitals which operating this patients in less than 48 h after admission, the Ministry of Health should consider expending the usage of these methods, not only for improving clinical outcomes, but also for reducing geographical variations in health services.
6. The Ministry of Health should measure and publish extensive data regarding the correlation between geographical erase and risk factors for cardiovascular disease and other disease.
7. The Ministry of Health should measure and publish extensive data regarding the distribution of medical practices by districts in order to explain the observed usage patterns or referral patterns.

## Conclusions

This study provides, for the first time, a broad comparison of health service utilization among areas in Israel, which can be divided into the center of the country and its periphery. The findings of this paper can help policy makers assess, plan and initiate strategies in service supply which will strengthen preventive medical services in the community, reduce cardiovascular risk factors in the periphery and expand the national publication of clinical quality scores.

## Additional files

Additional file 1: (PDF 180 kb)
Additional file 2: Cardiac catheterization service in the periphery, as a case study for health disparities reducing policy. (DOCX 14 kb)

## Abbreviations

NHDD: National Hospital Discharges Database
OECD: Organization for Economic Co-operation and Development
PTCA: Percutaneous transluminal coronary angioplasty

## Footnotes

1
http://www.dartmouthatlas.org

2 Note that “medical admissions” also excludes normal deliveries and day hospitalizations.

3
http://www.health.gov.il/PublicationsFiles/health2013.pdf

4
http://www.health.gov.il/PublicationsFiles/Quality_National_Prog.pdf
