# Supplementary material for: Geographic variation in selected hospital procedures and services in the Israeli health care system
Source: Isr J Health Policy Res. 2017 Jan 16;6:4. doi: 10.1186/s13584-016-0127-y (PMC5240306; doi:10.1186/s13584-016-0127-y)
Supplement: Additional file 1: — (PDF 180 kb) [file 13584_2016_127_MOESM1_ESM.pdf]

ANNEX A – CLASSIFICATION OF TERRITORIAL UNITS

Table 1. Classification of territorial units used for regional analysis at the OECD and examples of territorial units used in medical practice variation studies

Region	Territorial levels 2	Territorial levels 3	Examples of "small areas" in MPV studies
**Australia**	States/territories (8)	Statistical divisions (60)	Local Government Areas (LGAs) – administrative
**Austria**	Bundesländer (9)	Gruppen von Politischen Bezirken (35)	-
**Belgium**	Régions (3)	Provinces (11)	-
**Canada**	Provinces and territories (12)	Census divisions (288)	Local Health Integration Networks in Ontario (14) Agence de la santé et des services sociaux in Québec (18) Other similar regional health authorities in other provinces
**Chile**	Regions (15)	Provincias (54)	
**Czech Republic**	Oblasti (8)	Kraje (14)	-
**Denmark**	Regioner (5)	Landsdeler (11)	-
**Estonia**	Region (1)	Groups of maakond (5)	-
**Finland**	Suuralueet (5)	Maakunnat (20)	-
**France**	Régions (22)	Départements (96)	-
**Germany**	Länder (16)	Spatial planning regions (96)	-
**Greece**	Groups of development regions (4)	Development regions (13)	-
**Hungary**	Planning statistical regions (7)	Counties+Budapest (20)	-
**Iceland**	Regions (2)	Landsvaedi (8)	-
**Ireland**	Groups of regional authority regions (2)	Regional authority regions (8)	-
**Israel**	7 Districts		-
**Italy**	Regioni (21)	Province (107)	-
**Japan**	Groups of prefectures (10)	Prefectures (47)	-
**Korea**	Regions (7)	Special city, metropolitan area and province (16)	-
**Luxembourg**	State (1)	State (1)	-
**Mexico**	Estados (32)	Grupos de municipios (209)	-
**Netherlands**	Landsdelen (4)	Provinces (12)	-
**New Zealand**	Groups of regional councils (2)	Regional councils (14)	-
**Norway**	Landsdeler (7)	Fylker (19)	-
**Poland**	Vojewodztwa (16)	Podregiony (66)	-
**Portugal**	Comissaoes de coordenaçao e desenvolvimento regional + regioes autonomas (7)	Grupos de municipios (30)	-
**Slovak Republic**	Zoskupenia krajov (4)	Kraj (8)	-
**Slovenia**	Kohezijske regije (2)	Statistične regije (12)	-
**Spain**	Comunidades autonomas (19)	Provincias (59)	Health care areas (164)
**Sweden**	Riksomraden (8)	Län (21)	-
**Switzerland**	Grandes regions (7)	Cantons (26)	Hospital service areas (HAS, 83) – catchment area
**Turkey**	Regions (26)	Provinces (81)	-
**United Kingdom**	Government office regions + counties (12)	Upper tier authorities or groups of lower tier authorities or groups of unitary authorities or LECs or groups of districts (133)	Primary Care Trusts (PCTs) (151) – administrative
**United States**	States (51)	Economic areas (179)	Hospital Referral Regions (HHR, 306) – catchment area

ANNEX B – DETAILS ON PROCEDURE CODES

28. This section provides guidance on the first priority list of procedures/activities and second optional list. When possible, procedure codes and the sources used in their identification are provided using the Classification of Procedures of the ICD-9-CM[2]. Country experts that do not use this classification are invited to check with the Secretariat or with colleagues whether a mapping of ICD-9-CM with their own classification of procedures has already been done in the context of the participation to other OECD projects (annual data collection or hospital price comparison projects) or to other international projects (such as the ECHO project[3]).

29. For each procedure, rules for exclusion and inclusion are provided to standardise as much as possible the procedures/activities. The unit of analysis used to calculate the rates is included along with the suggested age group. Information on the optional variables is also provided and **any of these variables can be included where possible.**

First priority list

Hospital medical admissions

30. Experts agreed to report medical practice variations in hospital medical admissions (or discharges), as an indicator of the intensity of care delivered in territorial units. Countries should consider for inclusion any hospital inpatient stay (i.e., with at least one night) with a medical (non-surgical) purpose in a "hospital", as defined by the category HP.1.1(general hospitals) and HP. 1.3 (specialised hospitals) in the revised System of Health Accounts[4]. This category does not include mental hospitals or long- term care facilities. Where DRG-like classifications are used, medical admissions can be identified by medical (i.e. non surgical) DRGs, with an overnight stay.

31. Optional variable of the density of hospital beds is suggested as a resource use variable.

---

2 The International Classification of Diseases, 9th Revision, Clinical Modification" (ICD-9-CM), Sixth Edition, issued for use beginning October 1, 2008 for federal fiscal year 2009 (FY09). The ICD-9-CM is maintained jointly by the National Center for Health Statistics (NCHS) and the Centers for Medicare & Medicaid Services (CMS).

3 Countries participating in both the hospital price comparison project and the medical practice variations project are Australia, Belgium, Canada, Czech Republic, Finland, France, Germany, Italy, Netherlands, Spain, Sweden, Switzerland, United Kingdom and the United States. Countries participating in the ECHO project are: Austria, Denmark, Ireland, Portugal, Slovenia, Spain, and the United Kingdom.

4 See http://www.oecd-ilibrary.org/social-issues-migration-health/a-system-of-health-accounts_9789264116016-en, pp.130-133

Hospital Medical Admissions

**Description**	Hospital admission for a minimum one night inpatient stay. Hospitals are defined to be general or specialised hospitals (HP.1.1. and HP1.3 in the System of Health Accounts).
**Rules**	All **medical** discharges
**Exclusion**	Day care is not included. Exclude surgical discharges.
**Unit to be used for rates**	Per 100,000 population
**Age group (suggested) for women and men**	15-34, 35-44, 45-54,55-64,65-74,75+ OR 5-year age groups
**Resource use (optional)**	Density of hospital beds by territorial unit

Caesarean sections

32. Experts identified caesarean sections for the medical practice variations project as there is an overall rising trend on caesarean sections across the majority of OECD countries. Countries should consider all procedures where a baby is delivered by caesarean. These procedures can either be planned where the procedure becomes apparent during pregnancy, unplanned or an elective procedure on the basis of personal choice. The ICD-9-CM codes are provided below. Crude and standardised rates are commonly reported per 1,000 live births and will be the relevant unit for this procedure across a range of suggested age groups.

33. Optional variables include the average length of stay as a proxy measure of resource use and it has the advantage that it can be identified as the difference between date of discharge and date of admission. The density of obstetricians is suggested as the second resource variable.

Caesarean Sections

**ICD-9-CM code**	74.0-74.2 Classical , low cervical or extraperitoneal caesarean<br>74.4 Caesarean section of other specified type<br>74.99 Other caesarean section of unspecified type
**Rules**	Any procedure code
**Unit to be used for rates**	Per 1,000 live births
**Age group (suggested) for women**	<19, 20-24,25-29,30-34,35-39,40+ OR 5 year age groupings
**Resource use (optional variable)**	Average length of stay for a caesarean section by territorial unit
**Resource use (optional variable)**	Density of obstetricians by territorial unit

Revascularisation

34. Experts agreed on three procedures to study revascularisation: coronary bypass (CABG), coronary angioplasty (PTCA); and catheterization (diagnostic procedure). It is optional for countries to include catheterisation in this analysis.

35. The CABG procedure is used to divert blood around narrow or clogged arteries (blood vessels). This procedure involves taking a blood vessel from another part of the body (usually chest or leg) to use as a graft to replace any hardened or narrowed arteries to the heart. PTCA procedure is used to widen the blood vessel to increase blood flow to the heart. Catheterisation is the insertion of a catheter into a chamber

of the heart to assess the heart's condition as well as for interventional purposes. The ICD-9-CM codes are provided below.

36. To avoid double counting procedures for which more than one code may be used depending on each national classification system, only one code should be reported per procedure category for each patient. For example, if a percutaneous coronary intervention including a coronary stenting is recorded as two separate codes, only one code/procedure should be reported. Crude and standardised rates are suggested to be reported per 100,000 of the population in the territorial unit across age groups/gender. Data should be reported separately for each procedure.

37. Optional variables include average length of stay for CABG, and density of cardiac surgeons. Two need proxy variables are suggested and include the prevalence of heart disease as well as the mortality rate of coronary heart disease.

Revsacularisation

**ICD-9-CM code Coronary bypass**	36.1, 36.11-36.19 Aortocoronary bypass for heart revascularization
**ICD-9-CM code Percutaneous coronary interventions (PTCA and stenting)**	36.0 Removal Of Coronary Artery Obstruction And Insertion Of Stent(s)
**ICD-9-CM code Cardiac catheterisation (optional)**	37.21 Right Heart Cardiac Catheterization<br>37.22 Left Heart Cardiac Catheterization<br>37.23 Combined Right And Left Heart Cardiac Catheterization
**Rules**	Any principal diagnosis code. To avoid double counting procedures only one code should be reported per procedure category for each patient.
**Unit to be used for rates**	Per 100,000 population in the territorial unit
**Age group (suggested) for women and men**	20-49,50-64,65-74, 75+ , OR 5 year groups
**Resource use (optional variable)**	Average length of stay by territorial unit for coronary bypass
**Resource use (optional variable)**	Density of cardiac surgeons by territorial unit
**Need proxy (optional variable)**	Prevalence of ischemic heart disease by territorial unit (age/sex standardisation optional)
**Health outcome (optional variable)**	Direct standardised mortality rate of coronary heart disease by territorial unit

Knee interventions

38. Two knee interventions were agreed upon: knee replacement and knee arthroscopy (diagnostic procedure). It is optional for countries to include knee arthroscopy in this analysis.

39. In knee replacement surgery, the knee is replaced with an artificial joint because it is damaged (e.g. severe arthritis). The knee can be completely or partially replaced. Knee arthroscopy is a procedure performed through small incisions in the skin to repair injuries to tissues, cartilage or bone. An arthroscope is a small instrument guided by a lighted scope attached to a television monitor. The ICD-9-CM codes are provided below.

40. Crude and standardised rates are suggested to be reported per 100,000 of the population in the territorial unit across age groups/gender. Data should be reported separately for each procedure.

41. Optional variables include average length of stay for knee replacement, and density of orthopaedic surgeons. A pre-operative score is suggested as a proxy for need where available (e.g. EQ-5D Index score). The EQ-5D is a standardised instrument and has been used in medical practice variation studies to assess a patient's health status. A post-operative score is suggested as a health outcome variable. Some measures used in the literature include the EQ-5D Index score, Oxford Knee score, and SF-36. The Oxford Knee score is another survey instrument designed to capture information on pain/mobility. The SF-36 is a measure used to estimate disease burden in a number of disease areas including musculoskeletal conditions.

Knee Interventions

**ICD-9-CM code Knee replacement**	81.54 Total knee replacement<br>81.55 Revision of knee replacement, not otherwise specified<br>OR 00.80-00.84 Revision of knee replacement if specified
**Rules knee replacement**	Any principal code
**Inclusion knee revision**	Revision of knee replacement
**Knee arthroscopy (optional)**	80.26 Arthroscopy knee and<br>80.6 Excision of semilunar cartilage of knee
**Rules knee arthroscopy**	Only one code should be reported per event/patient.
**Unit to be used for rates**	Per 100,000 population
**Age group (suggested)**	15-34,35-44,45-54,55-64,65-74,75+ OR 5 year age groups
**Resource use (optional variable)**	Average length of stay for knee replacement by territorial unit
**Resource use (optional variable)**	Density of orthopaedic surgeons by territorial unit
**Need proxy on pain/mobility (optional variable)**	Pre-operative score by territorial unit for knee replacement (e.g. EQ-5D Index score)
**Health outcome on pain/mobility (optional variable)**	Post-operative score for knee replacement by territorial unit (e.g. Oxford Knee Score, EQ-5D Index score, SF-36)

Surgery after hip fracture
(Low variation procedure for calibration purposes)

42. Surgery after hip fracture was proposed for calibration purposes as this procedure is reported to have low variation in the literature. A number of procedures exist for the treatment (e.g. total hip replacement, partial replacement, the use of nails/screws). The ECHO project is also studying variations in surgery after hip fracture which considers all forms of repair. The Secretariat proposes to adopt this approach. Below is a list of codes in ICD-9-CM kindly provided by investigators of the ECHO project. All hip fracture emergency admissions are included regardless of the way in which the hip was repaired. This measure is a proxy for the burden of disease for hip fracture because treatment is typically provided for this condition. External causes are excluded (e.g. accidents).

43. Crude and standardised rates are suggested to be reported per 100,000 of the population in the territorial unit across age groups/gender.

44. Optional variables include average length of stay. A pre-operative score is suggested as a proxy for need where available (e.g. EQ-5D Index score). A post-operative score is suggested as a health outcome variable. Some measures used in the literature include WOMAC, EQ-5D Index score, and SF-36. The WOMAC score is survey instrument to capture information on pain/mobility after hip surgery.

Surgery after hip fracture

**ICD-9-CM code**	820.0-820.3, 820.8,820.9 Only emergency admissions of fracture of neck of femur<br>Plus 733.14 Pathologic fractures
**Rules**	Principal diagnosis code (Emergency admission) can be reported with or without the pathologic fractures.
**Exclusion**	E800-E849.9 (Accidents: railway, motor vehicle, road, water, air and space)
**Unit to be used for rates**	Per 100,000 population in the territorial unit
**Age group (suggested)**	15-34,35-44,45-54,55-64,65-74,75+ OR 5 year age groups
**Resource use (optional variable)**	Average length of stay by territorial unit
**Need proxy on pain/mobility (optional variable)**	Pre-operative score by territorial unit (e.g. EQ-5D Index score)
**Health outcome on pain/mobility (optional variable)**	Post-operative score (e.g.WOMAC, EQ-5D Index score, SF-36) by territorial unit

Second optional list

Hysterectomy

45. The Secretariat proposes to consider all types of hysterectomies, be they partial or complete, abdominal or vaginal. The table below shows procedures codes in ICD-9-CM. All diagnoses should be included. The unit of analysis for rates is the number of procedures for 1,000 of the female population.

Hysterectomy

**ICD-9-CM code**	68.3-68.9 Abdominal or vaginal hysterectomy
**Rules**	Any principal diagnosis code
**Unit**	Per 1,000 female population in the territorial unit
**Age group (suggested)**	15-34,35-44,45-54,55-64,75+ OR 5 year age groups
**Resource use (optional variable)**	Average length of stay by territorial unit
**Resource use (optional variable)**	Mean waiting time by territorial unit

MRI exams

46. The availability of MRI exams has increased rapidly in most OECD countries. Experts agreed that it may be useful to examine the degree of geographical variation in the use of diagnostic tests such as MRI exams.

47. The variable of interest is the number of patients receiving the exam. Crude and standardised rates are suggested to be reported per 100,000 of the population in the territorial unit across age groups/gender.

48. The optional variable suggested is the number of MRI machines per capita.

MRI Exam

**Unit to be used for rates**	Number of patients receiving MRI exams per 100,000 population in the territorial unit
**Age group (suggested)**	15-34,35-44,45-54,55-64,65-74,75+ OR 5 year age groups
**Resource use (optional variable)**	Number of MRI machines per capita

CT exam

49. The second diagnostic test is CT exam which has also increased rapidly in most OECD countries.

50. The variable of interest is the number of patients receiving the CT exam. Crude and standardised rates are suggested to be reported per 100,000 of the population in the territorial unit across age groups/gender.

51. The optional variable suggested is the number of CT machines per capita.

CT Exam

**Unit to be used for rates**	Number of a patients receiving the CT scan per 100,000 population in the territorial unit
**Age group (suggested)**	15-34,35-44,45-54,55-64,65-74,75+ OR 5 year age groups
**Resource use (optional variable)**	Number of CT machines per capita
