# Supplementary material for: Geographic variation in selected hospital procedures and services in the Israeli health care system
Source: Isr J Health Policy Res. 2017 Jan 16;6:4. doi: 10.1186/s13584-016-0127-y (PMC5240306; doi:10.1186/s13584-016-0127-y)
Supplement: Additional file 2: — Cardiac catheterization service in the periphery, as a case study for health disparities reducing policy. (DOCX 14 kb) [file 13584_2016_127_MOESM2_ESM.docx]

**Appendix 2**

**Cardiac catheterization service in the periphery, as a case study for health disparities reducing policy**

Tracing trends in the field of cardiac catheterization between the years 2002-2012 indicates that there have been targeted intentional and non-intentional interventions to improve this service in the periphery. We will focus briefly on two major cardiologic interventions that were implemented by the Ministry of Health in the periphery. The first relates to intentional interventions in infrastructures and manpower, in order to guarantee essential health services. These interventions included building and renovating old and new cardiac catheterization units. An additional intervention was based on the policy of creating incentives to encourage "change agents" in needed areas. In this specific case these "change agents" were trained cardiologists and the incentives were based on financial benefits and grants that were given to cardiologists who move to the periphery, especially between the years 2000-2005.

The second relates to non-intentional interventions. An examination of the entire cardiology spectrum in Israel reveals that the overpricing of the cardiac catheterization procedure contributed to the dramatic increase in the performance of this procedure19. In our opinion, this overpricing caused a chain reaction that started by encouraging all the hospitals to expand their cardiac catheterization services, especially in the periphery, places where this procedure was not as developed as in the rest of Israel. Extra resources triggered accelerated development in this field and attracted many young doctors to seek training causing an excess supply of skilled cardiologists specializing in cardiac catheterization. This national overflow of skilled personnel encouraged migration of cardiologists to the periphery.

However, according to this assumption and given the processes described above, we could expect the upward trend in the field of Interventional Cardiology in the periphery to cause performance rates to match rates in the rest of the country. In practice, these processes have created a significant gap in the incidence, stated above, of catheterization in the periphery compared to the Central district (1.5 times higher). It might be that the high catheterization rate in the periphery in the last 12 years represents compensation for under treatment in the years before although the gap may remain constant because of higher morbidity rates among the population in these areas. On the other hand, given the incentives mentioned above, one could wonder whether there was an excessive performance of catheterization.
